# Detection of Parasites in the Field: The Ever-Innovating CRISPR/Cas12a

**DOI:** 10.3390/bios14030145

**Published:** 2024-03-14

**Authors:** Xin Li, Zhisheng Dang, Wenqiang Tang, Haoji Zhang, Jianwei Shao, Rui Jiang, Xu Zhang, Fuqiang Huang

**Affiliations:** 1 School of Life Science and Engineering, Foshan University, Foshan 528225, China; lixin990730@163.com (X.L.);; 2 National Institute of Parasitic Diseases, Chinese Center for Diseases Control and Prevention (Chinese Center for Tropical Diseases Research), Key Laboratory of Parasite and Vector Biology, National Health Commission of the People’s Republic of China (NHC), World Health Organization (WHO) Collaborating Center for Tropical Diseases, National Center for International Research on Tropical Diseases, Shanghai 200025, China; 3 State Key Laboratory of Hulless Barley and Yak Germplasm Resources and Genetic Improvement, Lhasa 850002, China; 4 Tibet Academy of Agriculture and Animal Husbandry Sciences, Lhasa 850002, China; 5 College of Animal Science and Veterinary Medicine, Huazhong Agricultural University, Wuhan 430070, China

**Keywords:** detection, CRISPR, Cas12a, suboptimal crRNA, light-activated crRNA, tandem repeats, POCT

## Abstract

The rapid and accurate identification of parasites is crucial for prompt therapeutic intervention in parasitosis and effective epidemiological surveillance. For accurate and effective clinical diagnosis, it is imperative to develop a nucleic-acid-based diagnostic tool that combines the sensitivity and specificity of nucleic acid amplification tests (NAATs) with the speed, cost-effectiveness, and convenience of isothermal amplification methods. A new nucleic acid detection method, utilizing the clustered regularly interspaced short palindromic repeats (CRISPR)-associated (Cas) nuclease, holds promise in point-of-care testing (POCT). CRISPR/Cas12a is presently employed for the detection of *Plasmodium falciparum*, *Toxoplasma gondii*, *Schistosoma haematobium*, and other parasites in blood, urine, or feces. Compared to traditional assays, the CRISPR assay has demonstrated notable advantages, including comparable sensitivity and specificity, simple observation of reaction results, easy and stable transportation conditions, and low equipment dependence. However, a common issue arises as both amplification and cis-cleavage compete in one-pot assays, leading to an extended reaction time. The use of suboptimal crRNA, light-activated crRNA, and spatial separation can potentially weaken or entirely eliminate the competition between amplification and cis-cleavage. This could lead to enhanced sensitivity and reduced reaction times in one-pot assays. Nevertheless, higher costs and complex pre-test genome extraction have hindered the popularization of CRISPR/Cas12a in POCT.

## 1. Introduction

Parasitosis, which is infection with parasites, is a prevalent cause of morbidity among humans worldwide [1,2,3]. Tropical zones, particularly those that are impoverished, conflicted, or unsanitary, serve as endemic foci for a range of parasitic diseases [3,4,5]. The World Health Organization (WHO) has reported that annually 48.4 million cases and 59,724 deaths are attributed to the prevalence of 14 parasites, accounting for a total burden of 8.78 million disability-adjusted life years (DALYs). Of these, 48% represent foodborne parasitic diseases, accounting for 76% of the DALYs [6]. Transmission through contaminated food is prevalent in low- and middle-income countries [6]. Approximately 241 million cases of malaria and 627,000 deaths resulting from malaria were reported globally in 2020. Innumerable deaths are caused by other parasitic infections, most notably neglected tropical diseases (NTDs) [2,5,7].

Unlike the vast majority of bacterial and viral infections, which have an incubation period ranging from a few hours to days, parasitic diseases tend to have an incubation period of weeks or even months. The incubation period of specific parasitic diseases, like alveolar echinococcosis, can extend up to 10 years [2,8]. Therefore, early and precise detection of parasitosis is imperative for timely curative interventions and prevention of pandemics. However, current reliable or commonly used detection methods are limited by sensitivity, reaction time, and equipment dependence to achieve this purpose [9,10].

A promising new method for nucleic acid detection utilizes the CRISPR-associated (Cas) nuclease, which can overcome the limitations of instrument dependence and laborious operational processes [11,12,13,14,15]. By meticulously selecting target genes and designing specific CRISPR crRNAs (crRNAs), precise detection of various parasites can be guaranteed [13,15,16,17]. Due to the precise recognition capability of crRNA, a broad operational temperature range, and an intuitive result observation method, it is progressively evolving into an optimal tool for on-site testing [18,19,20,21]. When used with thermostatic amplification techniques like RPA and LAMP, CRISPR/Cas12a shows higher specificity and sensitivity [22,23,24,25,26,27]. The main objective of testing in field environments is to decrease the number of devices and simplify transportation conditions while maintaining both specificity and sensitivity. To improve the applicability and effectiveness of parasite monitoring in the field, continuous optimization of existing one-pot detection systems and the development of convenient biosensors that combine all essential steps into one are crucial.

The present review compares the CRISPR/Cas12a system with alternative molecular methods for the detection of parasitic diseases. Emphasis is on enhancement of the one-pot recombinase polymerase amplification (RPA)-CRISPR/Cas12a and improvement of CRISPR/Cas12b or Cas13 assays.

## 2. Application of Nucleic Acid Amplification Tests in Parasite Detection

Currently, the diagnosis of parasitic diseases relies on several approaches, such as epidemiology and pathophysiology, and methods including microscopy, immunodiagnostics, and nucleic acid amplification tests (NAATs). Among these, the microscopic detection of parasites remains the most reliable [9,28]. However, in underdeveloped regions with high rates of parasitosis, skilled microscope operators are often scarce, making this technique challenging to implement [29]. Furthermore, this approach is unsuitable for conditions linked to parasites at different developmental stages, which pose challenges in their detection within blood or stool specimens.

Immunoassay-based diagnostic procedures have been used for decades and are widely used for detecting parasites. However, their application for diagnosis of parasitosis has been limited due to several drawbacks including the possibility of false negatives and false positives [30,31,32].

Molecular detection of nucleic acids demonstrates superior sensitivity, specificity, and reproducibility compared to alternative methods (Table 1). Consequently, NAATs are preferred molecular detection tools due to their ability to amplify trace amounts of DNA and RNA, allowing for highly specific detection by complementary nucleotide pairing [2,9]. Polymerase chain reaction (PCR) is currently the most prevalent NAAT tool. Among them, quantitative real-time PCR (qPCR) has demonstrated the best sensitivity and specificity in the detection of various parasites and digital PCR (dPCR) can be powerful in quantifying nucleic acids [2,33,34,35,36]. Furthermore, a PCR-ELISA-based detection technique has been established, reducing the limit of detection (LOD) to 0.3 fg, equivalent to 0.004 parasites; however, this method takes longer than 4 h [37]. Although these techniques have been instrumental in establishing dependable diagnostic methods for parasitosis, including malaria, filariasis, toxoplasmosis, and echinococcosis, they require prolonged reaction times, intricate handling, expensive laboratory equipment, and a high level of technical expertise [10,38,39,40,41].

Isothermal amplification has been employed in the diagnosis of various parasitic diseases, addressing the challenges posed by traditional diagnostic methods [42,43,44,45,46]. Compared to PCR, isothermal amplification technology, exemplified by loop-mediated isothermal amplification (LAMP) and RPA, significantly reduces the reaction time and dependence on instruments. RPA is an efficient method for on-site detection due to its simple primer design, low-temperature requirements, and easy storage [43,44]. Recombinase-aid amplification (RAA), based on the same principle, also offers these advantages in rapid detection [47]. The assay results presentation has transitioned from gel electrophoresis to using fluorescence, turbidity, color, and lateral flow, which are easier to manipulate and observe, thus enhancing their field operation applicability [42,48,49].

In various laboratories and regions, qPCR remains the primary or sole standard in NAAT due to issues with standardizing other assays such as PCR, RPA, and LAMP [50]. Determining the reliability of results and setting a reliable assay time are among the challenges. Sequencing is a common method of validation, but it significantly extends the time required to obtain assay results. Environmental factors, such as temperature and humidity, may also limit the application of these technologies for field monitoring by affecting the stability and reliability of the reagents. To address this issue, sealed lyophilized powders can be used to preserve the reagents [20].

In addition to the selection of appropriate detection methods for NAATs, which has a significant impact on the accuracy and sensitivity of diagnosis of parasitosis, the selection of target genes is a key consideration. Along with 18S ribosomal RNA (rRNA), Internal Transcribed Spacer (ITS), and mitochondrial genes, stable tandem repeats have recently come into focus. In most parasite genomes, repetitive sequences make up a substantially greater proportion compared to coding sequences, comprising an estimated 20% or even surpassing 30% [51,52]. Numerous tandem repeats have been used to detect multiple protozoans and worms, such as *Trypanosoma cruzi*, *Onchocerca volvulus*, and *Schistosoma mansoni* (Table 2).

Point-of-care testing (POCT), a priority for strategies relying on mass drug administration to control several NTDs, is a medical diagnostic tool that can be used near or at the point-of-care, allowing for on-site testing [20,76]. In ideal POCT, the steps required to go from raw sample to understandable result should be minimized, enabling unskilled operators to perform the analysis. Therefore, it is imperative to develop nucleic acid-based diagnostic tools that combine the sensitivity and specificity of established NAATs with the convenience, cost-effectiveness, and speed of isothermal amplification-based POCT methods. CRISPR-based diagnostics have the potential to fulfill all these requirements (Table 3).

## 3. CRISPR/Cas12a for POCT

### 3.1. Discovery of CRISPR

The CRISPR/Cas system was originally discovered by Ishino in 1987 [77] and officially named as such in 2002 [78]. Subsequently, there has been extensive research focusing on the identification and characterization of the proteins and molecules associated with the CRISPR/Cas system [79]. CRISPR/Cas systems are composed of Cas genes organized in operons and a CRISPR array, which comprises unique genome-targeting sequences (called spacers) interspersed with identical repeats [80]. These systems exhibit some unprecedented advantages, including rapid and accurate gene recognition [80,81] and reaction temperature under physiological conditions [82,83,84]. Jennifer Doudna and Emmanuelle Charpentier were the first to illustrate the potential of the CRISPR/Cas9 system as a means of gene editing [80]. CRISPR/Cas9 is not only the first discovered CRISPR gene editing tool [80,85,86], but also the first CRISPR-based diagnostic tool [87,88]. Subsequently, Janice Chen and Feng Zhang played pivotal roles in the primary investigations of CRISPR/Cas12a [82] and CRISPR-Cas13a [89], particularly in the context of applications in detection.

### 3.2. CRISPR/Cas12a System

The Cas12a effector protein, also referred to as the Cpf1 effector protein, is a programmable RNA-guided DNA nuclease that was identified as part of the type-V class II CRISPR-Cas system [90,91]. This protein may be associated with a distinct TnpB transposase gene family [92]. Cas12a has a bilobed architecture consisting of an N-terminal recognition lobe (REC) and a C-terminal nuclease lobe (NUC) connected by the wedge (WED) domain [93]. The REC lobe binds crRNA, while the NUC lobe contains the PAM-interacting (PI), bridge helix (BH), RuvC, and Nuc domains [93,94]. In comparison to Cas9, the design of the Cas12a system is simpler and more cost-effective since it only requires one crRNA and no trans-activating crRNA (tracrRNA) [17]. A tool has been developed that enables the rapid design of highly specific CRISPR/Cas12 crRNA [95].

The CRISPR/Cas12a system has also been applied to gene editing [96,97], with a current focus on nucleic acid detection [98,99]. Cas12a accurately identifies single-stranded DNA (ssDNA) and double-stranded DNA (dsDNA), creating gaps by recognizing T-rich protospacer adjacent motif (PAM) sequences and catalyzing its crRNA maturation (ssDNA activator needs no PAM sequence) [17,82,93]. It shows higher tolerance for mismatches and lower specificity when targeting ssDNA compared to dsDNA [82,100]. It was discovered that Cas12a also exhibits collateral activity and can cleave ssDNA without the presence of a complementary crRNA sequence [82,93,101]. The non-target strand and RuvC domains are highly flexible, with the target strand being particularly flexible when located at the nuclease active site. Consequently, the RuvC domain becomes significantly active during R-loop formation, enabling the entry of ssDNA into the active center of the enzyme, for degradation [102]. In addition, this cis-recognition-triggered trans-cleavage presents a multiple turnover behavior [82,103]. This feature enables the Cas12a system to have a robust signal amplification mechanism [81,82,93,101], prompting the establishment of a new type of CRISPR diagnostic assay [84,104,105,106,107,108].

**Figure 1:**
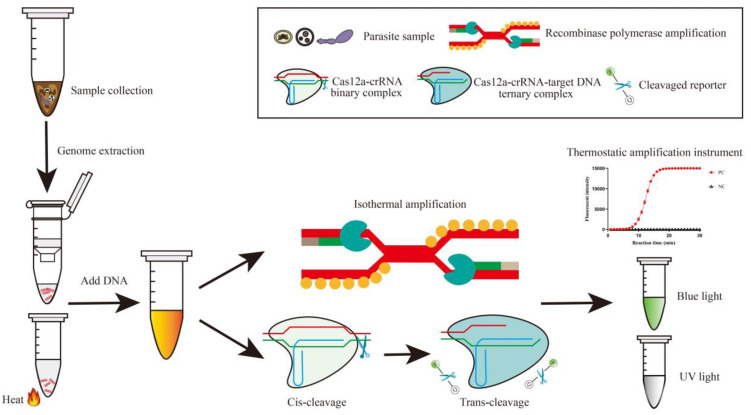
Scheme of CRISPR/Cas12a one-pot detection assay for parasites. Total DNA is extracted from samples containing parasite eggs or tissue fragments using either heat/vortex lysis or silica gel column chromatography. The DNA undergoes processing in a tube that contains a thermostatic amplification and CRISPR/Cas12a reaction system. Positive signals are generated when probes are cleaved by activated Cas12a under the target gene sequence recognized by the crRNA, resulting in the release of intuitive fluorescence under blue or UV light or in a thermostatic amplification instrument.

Therefore, the target DNA has the potential to act as an activator, triggering both cis- and trans-cleavage events of the Cas12a nuclease. The fluorophore/quencher-labeled (FQ) ssDNA reporter in the system is then cleaved, releasing a fluorescence signal that is measured to detect the pathogen (Figure 1). In Cas12a-based fluorescent biosensors, the length of the FQ ssDNA reporter is also an important factor affecting the sensing performance [109]. Reporter genes with 8 nt may be optimal for detection to avoid decreased efficiency of fluorescence caused by excessively long or short ssDNA reporters [109,110]. In addition to the fluorescence output mode, various types of detection methods such as lateral flow analysis (LFA) [25,111,112] and magnetic pull-down-assisted colorimetric method [113] have been developed in order to further reduce the dependence on the instrument used to observe the results. The CRISPR/Cas12a system has found extensive application in the detection of COVID-19, with studies demonstrating a detection limit as low as 5–10 copies [111,114,115,116,117]. The sensitivity of the method is comparable to that of qPCR and results are obtained via naked-eye observation within 45 min. Careful consideration of the mismatch location can also enable highly specific detection of various COVID-19 variants [118]. These advantages enable the detection of parasites through the CRISPR/Cas12a system, offering benefits beyond those of other nucleic acid detection methods (Table 3).

Benefiting from the interdisciplinary research of materials, engineering, analytical chemistry, medicine, and numerous other disciplines, the CRISPR/Cas12a technology has undergone continuous innovation and development. Initially envisioned as a gene editing or pathogen detection tool, it is now being or expected to be applied in various fields, including nucleic acid quantification [119,120], diverse small molecules detection [106,108,121], protein detection assay [122,123], telomerase activity assays [124], RNA detection [121], and other CRISPR/Cas-based biosensors [125].

### 3.3. CRISPR/Cas12a for Rapid One-Site Detection

Various assays and sensors have been developed for on-site CRISPR-based detection [20]. With the assistance of various amplification techniques, the sensitivity, specificity, and time required of Cas12a assays have been significantly enhanced [109]. The RPA reaction is less specific because it is typically conducted at a lower temperature of 37–42 °C. However, this can be compensated by the process of target gene recognition through crRNA in the Cas12a assay, further enhancing reaction specificity. The collateral activity of Cas12a has also replaced the more complex method of observing the results of the RPA reaction. Furthermore, both reactions can be performed efficiently at the same constant temperature, in a simple instrument, making this combination highly suitable for POCT [20]. Additionally, other thermostable amplification methods, such as LAMP, can be combined with Cas12b at a higher temperature of 65 °C [126]. Even without pre-amplification of target genes, it is possible to detect lower concentrations by combining CRISPR/Cas12a with hybridization chain reaction (HCR) for amplification-free clinical diagnostics or agricultural screening [127].

In addition to its combination with isothermal amplification technology, Cas12a can also interact with other Cas enzymes. After activation by the target gene, the trans-cleavage of CRISPR/Cas12a cleaves all surrounding ssDNA indiscriminately, which limits the simultaneous detection of multiple pathogens. Through orthogonal Cas12a and Cas13a, dual-gene detection can be achieved by adding a dual-labeled ssDNA trans-cleavage substrate and a single-stranded poly (U) RNA probe to the detection system [128]. This method allows for the simultaneous detection of multiple genes from the same pathogen to improve accurate detection [129], as well as individual genes from multiple pathogens for multiple detections. In addition to CRISPR/Cas effector protein-based [128,130], there are two other strategies for CRISPR multiplex detection: signaling-based and segregation-based [131,132]. Future CRISPR multiplex assays present challenges in fundamental research and evaluating the stability of CRISPR/Cas systems [131].

Significant developments have also been made in the development of CRISPR/Cas12a-based POCT sensors [20,133]. According to the WHO, POCT must adhere to the ASSURED guidelines (Affordable, Sensitive, Specific, User-friendly, Robust and rapid, Equipment-free, Deliverable to all people who need the test) [20,134]. This requires that the CRISPR/Cas12a reaction system be stable under any environmental conditions and be integrated into a simple biosensor to allow non-specialists to perform and interpret the test. CrRNA can be stabilized upon binding to CRISPR/Cas12a effector proteins, especially in the lyophilized powder state, and thus can be used in combination with POC devices to form an efficient nucleic acid detector. The sensors developed to date have incorporated a range of readout mechanisms, including fluorescence [89,133,135,136], colorimetric [130,132,133,136,137,138,139,140], and electronic methods [104,141,142], ensuring system stability while improving the availability of test results. Nevertheless, achieving a balance between cost-effectiveness, quality, and convenience remains a challenge for these sensors [76]. After addressing challenges related to quantification, multiple detection, and target amplification, it is important to continuously optimize the CRISPR/Cas12a system and sensor materials to improve their applicability in resource-limited regions.

### 3.4. Application of CRISPR/Cas12a for Parasite Detection

The application of the CRISPR technique for diagnosing parasitic diseases, like malaria, has undergone thorough evaluation over the years. Asymptomatic carriers, with low parasitic load, considerably hinder the control and eradication of the parasite. Achieving malaria eradication mandates a hyper-sensitive diagnosis of infections with a low parasitic load [2]. Unfortunately, resource-limited areas experience frequent malarial outbreaks, posing a challenge to screening carriers. Lee and colleagues developed a nucleic acid diagnostic method to detect *Plasmodium falciparum* by combining CRISPR/Cas12a with RT-RPA [23].

The method involves heating human serum, whole blood, or dried blood spots in a buffer at 95 °C for 10 min, followed by the transfer of the suspended sample to a pre-mixed Cas12a-RPA system. The mixture is then incubated at 40 °C for 30 min. The reaction outcomes can be observed using a plate reader or a handheld fluorometer, facilitating on-site detection. This technique significantly lowers the LOD to 0.36 parasites per microliter, which is well within the WHO’s rapid diagnostic test threshold of 200 parasites per microliter [143].

Additionally, the CRISPR/Cas12a assays has been successfully utilized for detecting *Toxoplasma gondii* [24,25,144]. These detection systems achieved a sensitivity of at least 1.5 copies target genes per microliter, surpassing that of real-time fluorescent RPA (33 genome copies per microliter) and other comparable methods [145]. Furthermore, this system was utilized to examine a range of parasites, including *Schistosoma haematobium* [27], *Cryptosporidium parvum* [26,112], *Enterocytozoon hepatopenaei* [146], *Clonorchis sinensis* [147], and *Heterodera schachtii* [148] (Table 4). These examples all demonstrate that CRISPR/Cas12a has comparable specificity and sensitivity to traditional assays. Especially when combined with thermostatic amplification, both specificity and sensitivity are doubly guaranteed.

It is estimated that 47% of the global population lacks adequate access to medical diagnostic tools, particularly in underdeveloped regions [149]. Cas12a and RPA-based diagnostic technologies are anticipated to effectively address this challenge, as the underlying method aligns with the majority of POCT requirements [134]. Nevertheless, several concerns need to be addressed due to the relatively short duration of research and the limited scope of large-scale clinical trials. The improvement in sensitivity of these assays for POCT, particularly concerning specific sample preparation, requires attention. Moreover, there is a need to reduce the detection time to obtain results.

## 4. Optimization of the CRISPR/Cas12a One-Pot Detection Assay

Due to the low initial concentration of the target gene in a sample and kinetic rates that result in an amplification-free LOD in the picomolar range under standard assay conditions [150], Cas12a detection often requires an amplification process before application. This implies that the signal amplification is usually conducted in two processes [109]. Target genes are initially amplified using RPA or LAMP techniques. The resulting amplified products are subsequently transferred to the Cas12a system for cleavage, followed by fluorescence signal generation.

To streamline operations and prevent cross-contamination during field tests, the one-pot method is now predominantly utilized. This assay allows the simultaneous amplification and cleavage of Cas12a. Freeze-drying all components and integrating them into the sensor ensures consistent performance in various environmental conditions [27,125]. However, this leads to the cis-cleavage of Cas12a, which reduces the concentration of the target genes while RPA enhances it. Therefore, it is crucial to optimize amplification in the initial phase of the reaction.

POCT sensors that use amplification followed by detection strategy often require multiple liquid transfers during testing, which reduces their user-friendliness [20]. The CRISPR/Cas12a one-pot detection system shows promise in replacing reagents in more mature amplification-free POC sensors, which can further improve the sensitivity of detection. However, significant challenges remain in terms of cost and complexity of devices.

### 4.1. One-Pot One-Step Reaction

#### 4.1.1. Determinants of Cas12a Enzyme Kinetics

Several studies have reported rapid single-turnaround, cis-cleavage reactions at low target concentrations, with typical reaction times of approximately 100 s [151]. A Michaelis–Menten model for Cas12a trans-cleavage activity was established and validated by a team from Stanford University. This was achieved through the utilization of varying concentrations of substrates, targets, and crRNAs [150,151]. The authors suggest that the concentration of the trans-cleavage product formed over time can be described using the following scaling equation:

(1) $$\frac{\left[P\right]\left(t\right)}{S_{0}}\approx (1-\exp\left(-\frac{t}{\tau }\right))$$

**Figure 2:**
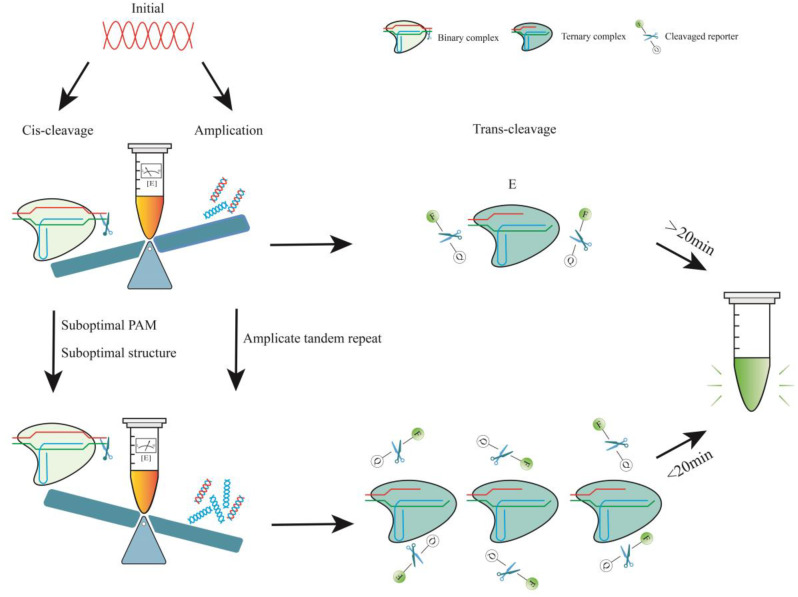
Effect of amplification and cleavage on detection efficiency. E is the target-activated Cas12-crRNA-target DNA complex. During the initial stage of the one-pot procedure, cis-cleavage is immediate, and the enzyme concentration is substantially greater than the target concentration. As amplification becomes more dominant, including instances when amplification efficiency is increased and cis-cleavage speed is reduced, the amount of [E] becomes substantially larger and can be rapidly increased, resulting in a significant improvement in reaction efficiency. Conversely, if the target is rapidly depleted in the initial stage, the emission of the fluorescent signal will decrease.

The production efficiency of the trans-cleavage product P is influenced by both reaction time and τ. To refer to the target-activated Cas12-crRNA-target DNA complex, we use E, and subsequently, [E] represents the concentration of this complex. The characteristic time required to complete trans-cleavage is governed by the time scale τ, which is proportional to *K_M_* and inversely proportional to *kcat* and [E] [151]. The rate constant *kcat*/*K_M_* of enzymatic reactions is affected by the Cas type, crRNA, incubation time, pH, and temperature [150]. During the early stages of the reaction, [E] equals the concentration of the target molecule (c), which depends on c_0_, amplification, and cis-cleavage. Therefore, one could use suboptimal crRNA to weaken cis-cleavage or employ other methods to ensure that amplification dominates the pre-reaction period, resulting in a rapid increase of [E] (Figure 2).

In the CRISPR/Cas12a system, the crRNA binds to the Cas12a effector protein to form a binary complex (ribonucleoprotein). This complex then locates the PAM sequence and verifies the adjacent spacer sequence, thereby initiating both cis- and trans-cleavage [91,94]. When using crRNAs with suboptimal structures or suboptimal PAMs, their cleavage activity may be impaired by affecting the efficiency of binding or recognition [22]. With these methods, amplicons can be rapidly accumulated for the activation of large numbers of Cas12a–crRNA–target ternary complexes (Figure 2). Sensitivity and detection time were significantly improved without compromising specificity. It is important to note that suboptimal crRNAs will also affect their collateral activity, thereby reducing the rate of fluorescence signal growth. Therefore, it is necessary to fully compare and screen the use of such suboptimal crRNAs.

#### 4.1.2. Reduced crRNA Efficiency by PAM

In the CRISPR-Cas system, the effector nuclease must identify the PAM adjacent to the target site for initiation of target recognition [152]. Studies of the crystal structure of the LbCas12a–crRNA binary complex [153] and AsCas12a–crRNA–target DNA ternary complex [94,154] reveal the mechanisms involved in Cas12a and crRNA recognition, as well as the operations of crRNA-directed DNA targeting and PAM recognition. In Cas12a, the PAM duplex is enveloped within a PAM-binding channel formed by the WED, REC1, and PI domains. The sequence and conformation of PAM duplexes are primarily recognized by two conserved lysine residues (i.e., base and shape readout mechanism) [155]. These findings suggest that the PAM-binding channel of Cas12a is flexible in conformation, allowing for the identification of both canonical and non-canonical PAMs [155]. LbCas12a and AsCas12a identify TTTV and CTTV/TCTV/TTCV as canonical and suboptimal PAM, respectively [155,156].

In 2022, a Chinese team conducted a one-pot test called sPAMC, which refers to a suboptimal PAM of a Cas12a-based test [22]. A comparison of the collateral activity revealed that crRNAs utilizing suboptimal PAMs demonstrated lower potency and slower kinetics in comparison to those utilizing canonical PAMs. Nevertheless, over 80% of the 120 suboptimal PAMs displayed quicker reactions than canonical PAMs in the one-pot reactions.

The appearance of the target amplicon was observed 2 min after the one-pot reaction utilizing suboptimal PAM, in contrast to the 8–10 min required for canonical PAM. Utilizing a suboptimal PAM with varied concentrations of Cas12a/crRNA ribonucleoprotein yielded steady kinetic curves, in contrast to reactions with traditional PAMs. In one-pot reactions, several uncommon PAMs (such as VTTV, TCTV, and TTVV) and some TRTV, TTNT, and YYYN PAMs (excluding TTTV) outperformed canonical PAMs. The SARS-CoV-2 diagnostic method established using suboptimal PAM demonstrates a sensitivity comparable to that of qPCR, with a reaction time of merely 15 min [22]. However, it seems that this approach is not applicable to AapCas12b [157]. This may be due to the PAM sequence of AapCas12b (TTN) being potentially less adaptable than that of LbCas12a (TTTV), and even a single nucleotide mutation within AapCas12b’s PAM could significantly impair its activity [157].

Substituting residues within the PAM-interacting domain of Cas enzyme can achieve a similar effect in adjusting its activity. This idea has been applied to Cas12b by the same team and proved to be effective [157].

#### 4.1.3. Reduced crRNA Efficiency by Structure

Suboptimal crRNAs can be selected based on their structure while ensuring specificity. If CRISPR/Cas9 cleavage is an energy-driven process, its efficiency relies substantially on nucleotide hybridization and changes in folding-free energy [158,159]. The stability of guide RNA (gRNA)-DNA for gRNAs exhibiting different efficiencies significantly varies. When local sliding is examined, an energy model accurately predicts the efficiency of gRNAs. In CRISPR/Cas12a, research has shown that the activity of the Cas12a system is positively correlated with the stable binding between the activator and the crRNA [160]. The structure of the single ssDNA activator has also been found to affect the Cas12a trans-cleavage activity [160,161]. Furthermore, engineering a hairpin secondary structure in the crRNA spacer region can greatly improve its specificity [162]. Therefore, it is crucial to consider the use of suboptimal crRNAs when developing a one-pot detection method. Additionally, the efficiency of the RPA-CRISPR/Cas12a one-pot and one-step reaction can also be enhanced by using crRNAs that are not restricted by the PAM sequence [117] or by reducing the dosage of Cas12a [163].

### 4.2. One-Pot Reactions with Two Steps

#### 4.2.1. Light-Activated crRNA to Initiate Cleavage

Control of chemical reactions through photocontrolled techniques can be achieved in a non-contact manner within seconds. This technology has been used extensively in both CRISPR/Cas9 research and practice [164,165,166], and has also been progressively refined for CRISPR/Cas12a detection [167]. Initially, the CRISPR/Cas12a system is blocked by a photo-cleaved linker containing crRNA to ensure optimal RPA performance. After amplification, the Cas12a detection system is activated via light to initiate trans-cleavage and produce fluorescence signals [167]. However, the constant optimization of the ratio between the photocleaved linker and crRNA, along with the compromised stability of the Cas12a-crRNA complex due to the lack of pre-binding of crRNA to Cas proteins, hinders the effectiveness of this approach.

The same group of researchers subsequently developed a novel CRISPR/Cas12a detection assay that uses 6-nitropiperonyloxymethyl-caged thymidine (NPOM-dt) to modify crRNA [168]. This method involves caging crRNA to prevent base pairing between itself and the target, rather than binding it to the Cas enzyme. Rapid activation can be attained by photoinduced decaying, which makes this approach simpler, faster, and more stable. It should be noted that optimization of the irradiation time and the number and position of NPOM may need reconsideration for different pathogens. In the context of POCT, challenges persist with reagent storage conditions, actual amplification time, and the portability of illumination devices.

#### 4.2.2. Physical Separation of the Two Processes

In addition to performing two reactions simultaneously in one tube, it is also possible to physically separate the two reaction systems in one tube, to allow for sequential progression [20,169,170,171]. The CRISPR/Cas12a reagents are spun down for cleavage after DNA amplification by leveraging the enhanced surface tension of the protein-containing liquid [172]. Initially, the RPA reaction takes place at the base of the tube, while CRISPR/Cas12a is positioned at the lid, separate from the reaction. After amplification for 20 min, the CRISPR/Cas12a reagent is briefly spun into the reaction mixture without opening the tube. The reaction continues, and the RPA amplicon activates the Cas12a nuclease to trans-cleave the fluorescent ssDNA-FQ molecule, resulting in a fluorescent signal. However, this method is cumbersome, particularly in the context of large-scale POCT.

**Figure 3:**
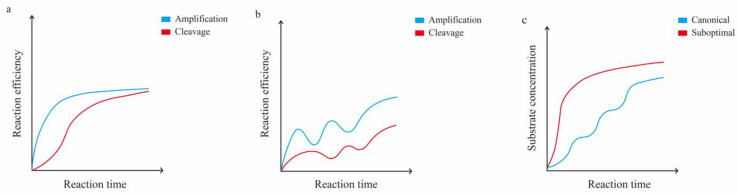
A schematic diagram of the relationship between amplification and cleavage in the CRISPR/Cas12a one-pot method. (**a**) Comparison of amplification and cleavage efficiency over time, using suboptimal protospacer or suboptimal crRNA to attenuate the early cleavage efficiency of Cas12a in the one-pot method. (**b**) Comparison of amplification and cleavage efficiency over time, using optimal crRNA in the one-pot method. (**c**) Variation in substrate concentrations over time when using suboptimal or optimal crRNA in the one-pot method.

In brief, cis-cleavage plays a crucial role as the rate-determining step for overall performance in one-pot reactions [22,151]. During the initial stage, low-concentration targets are diminished due to cis-cleavage, which results in a slow and unstable accumulation of amplicons. Consequently, the growth of the signal decreases or may even disappear altogether (Figure 3). The *kcat*/*K_M_* of the enzyme can be reduced by utilizing a suboptimal PAM or structure, which slows cis-cleavage. This results in a balance between the two-signal amplification processes of RPA and trans-cleavage. Through careful engineering of enzyme engineering [157,173], primer design, crRNA design [22,121,174,175], reaction system [125,130,174,176,177,178,179,180], and reporter selection [181,182], isothermal amplification and CRISPR detection can be effectively combined in a one-pot reaction [50]. The optimized Cas12a assay even has the potential to achieve the same detection performance at room temperature [157].

## 5. Conclusions

Parasites are prevalent in the natural world, particularly in underdeveloped regions, and result in high DALYs and substantial economic losses. This necessitates the development of rapid, sensitive, and accurate diagnostic tools for detecting parasites. The emergence of CRISPR, and specifically recent examinations of Cas12a, compensates for the limitations of isothermal amplification and presents a fresh approach for POCT. With the collateral activity of Cas12a, results can be evaluated intuitively via the inclusion of fluorophores. Combined with RPA, samples with even small numbers of pathogens can be quickly and accurately tested at the POC.

For POCT, the one-pot method is the best option due to its ability to prevent cross-contamination and the significant simplicity of the procedure. Nonetheless, current one-pot detection techniques are associated with several limitations, including extended reaction times, low sensitivity, complicated operation, and reliance on sample pretreatment. Additionally, the utilization of RPA has restricted the advancement of CRISPR assays to some extent. As the most commonly used partner for the CRISPR one-pot method, RPA kits are only sold by a few companies, with high prices and an unstable supply [48].

By balancing the two processes of amplification and cleavage with a suboptimal PAM or structure, the detection performance of the one-pot method can be improved. With suboptimal conditions, the limitation of PAM or crRNA on target genes can also be reduced, thereby expanding the pool of target genes. In addition, light-activated crRNA and spatial isolation enable two reactions to proceed in one-pot sequentially without the requirement for opening the lid. Furthermore, incorporating tandem repeats as targets can significantly enhance the amplification efficiency and sensitivity of detection, regardless of sample preparation methods. These modifications can potentially enhance not only RPA-Cas12a but also all Cas12a detection methods involving amplification. Furthermore, it is important to assess these concepts not only concerning Cas12a, but also in other CRISPR systems, including Cas12b, Cas13, and even Cas9.

The samples used for parasite testing are primarily blood and feces, and their nucleic acid extraction often relies on silica gel column chromatography that takes at least 45 min. In POCT, there is an increasing need for nucleic acid extraction methods that provide shorter operating times, simpler devices, and products with minimal inhibitors. Several POC nucleic acid extraction platforms have been developed to meet these requirements, including microfluidic chips, paper-based devices, microneedle patches, digital microfluidics, and hand-operated microfluidic systems [183]. However, these techniques are primarily focused on bacteria and viruses and have not been validated for parasite detection, and present challenges in extraction of high-quality RNA.

Although the sensitivity and accuracy of most CRISPR-based assays greatly exceed those of antigen test kits and isothermal amplification assays, which are currently more suitable for POCT, CRISPR still cannot replace them as the preferred choice for POCT, either due to their high cost or due to the complex storage and transportation conditions. In the future, for the application of CRISPR/Cas12a to POCT, it is necessary to continually optimize the one-pot method detection efficiency and identify a more compatible isothermal amplification technology. Alternatively, the existing amplification-free CRISPR/Cas detection technology can be further optimized [121,177,179,184].

Future studies should include larger-scale CRISPR/Cas12a clinical assay experiments and validation of sensor stability to ensure their effectiveness in various environments and conditions. Both artificial intelligence and machine learning are also expected to contribute to the rapid growth of the CRISPR system and parasite detection [185,186,187,188,189]. Academic institutions conduct research, industries engage in industrial production, and governments and organizations (e.g., WHO) allocate resources, invest, and establish ethical guidelines to ensure universal access to CRISPR/Cas12a diagnostics in low-resource settings where parasitic diseases are most prevalent.

## Figures and Tables

**Table 1:** Main strengths and weaknesses of different approaches for parasite detection.

Discipline	Strength	Weakness
Morphology	Accuracy (gold standard) Can detect multiple species at the same time	Lower sensitivity Difficulty distinguishing parasite-like egg High demand for professional skills
Immunology	Robust specificity Robust sensitivity	High cost and time consuming False positives for cross-reactivity False negatives in immunocompromised patients Inability to differentiate between ongoing and past infections
Molecular biology	Robust specificity Robust sensitivity Robust repeatability	High cost Limitations related to sample preparation and equipment Logistics systems requiring fresh sample analysis (e.g., cryogenic)

**Table 2:** List of partial parasitic repeat sequences.

Parasite	Repeat Sequence Name	Length (bp)	Quantity	GenBank Accession	Refs
**Protozoa**					
*Trypanosoma cruzi*	TCNRE	195	12% of the total genome	K01772	[53]
*Toxoplasma gondii*	/	529	200–300 copies per genome	AF146527	[54]
*Plasmodium falciparum*	Pfr364	716	41 copies per genome	/	[55,56]
*Plasmodium vivax*	Pvr47	333	14 copies per genome	/	[55,56,57]
**Cestodes**					
*Echinococcus granulosus*	EgG1 Hae III repeat	269	6900 copies per haploid genome (1% of *E. granulosus* genomic DNA)	DQ157697	[58,59]
*Taenia solium*	Tsol-9	158	/	U45987	[60]
*Taenia saginata*	HDP1	1272	0.4% of the *T. saginata* DNA	AJ133764	[61]
**Trematodes**					
*Schistosoma mansoni*	Sml-7 (DraI)	121	12% of the total genome	M61098	[62,63,64]
*Schistosoma haematobium*	DraI	121	over 15% of the *S. haematobium* genome	DQ157698	[65]
*Trichobilharzia ocellata*	ToSau3A	396	10,000 copies per haploid genome (1.5% of the *T. ocellata* genome)	AF442689	[66]
**Nematodes**					
*Strongyloides stercoralis*	/	765	/	AY028262	[67]
*Brugia malayi*	HhaI repeat	320	several thousand copies per haploid genome (about 12% of the genome)	M12691	[68,69]
*Wuchereria bancrofti*	SspI	195	300 copies per haploid genome	L20344	[70]
	LDR	1674	/	AY297458	[71]
*Onchocerca volvulus*	O-150	149	4500 copies per haploid genome	J04659	[72,73,74]
*Parafilaroides decorus*	Pd65	689	/	MT053285	[75]

Specific information on tandem repeats that have been used for parasite detection, including GenBank accession numbers and references, is provided. /: No data available.

**Table 3:** Comparison of CRISPR/Cas12a and commonly used detection technologies in molecular biology.

Technology	Device Dependency	Specificity	Reaction Time (min)	Number of Primers	Quantification	Cost	Results View Method	POCT Potential
PCR	Moderate	Robust	60–180	2	No	High	Gel electrophoresis	Moderate
qPCR (RT-qPCR)	High	Robust	>60	2	Yes	Extremely high	Fluorescent and computer system	LOW
dPCR	High	Robust	>60	2	Yes	Extremely high	Fluorescent and computer system	LOW
LAMP	Low	Robust	<60	4–6	No	Low	Gel electrophoresis Color Turbidity	High
RPA/RAA	Low	Moderate	20–60	2	No	Low	Gel electrophoresis Fluorescent Lateral flow	High
Cas12a	Low	Robust	20–60	2	No	Low	fluorescent Lateral flow	High

**Table 4:** Application of the CRISPR/CAS12 system to parasite detection.

Species	Method	Time (min)	LOD	Sample	Refs
*Plasmodium falciparum*	Cas12a-RPA	30 (+10) ^a^	0.36 parasites/μL	Serum/Whole blood/ Dried blood spot	[23,136]
*Plasmodium vivax*	Cas12a-RPA	30 (+10) ^a^	1.2 parasites/μL		
*Toxoplasma gondii*	Cas12a-RPA (two steps)	30 + >15	1.5 copies/μL	Whole blood	[25]
	Cas12a-RPA	35 (+20) ^a^	99~115 copies/μL	Environmental samples (e.g., water and soil)	[24]
	Cas12a-RAA (two steps)	20 + 50	1 fM		[144]
*Schistosoma haematobium*	Cas12a-RPA	40 + (70) ^a^	2 eggs	Urine	[27]
*Cryptosporidium parvum*	Cas12a-RPA (two steps)	30 + 60 (+20) ^a^	10 oocysts	Feces	[112]
	Cas12a-RPA	90	1 oocyst	Water	[26]
*Enterocytozoon hepatopenaei*	Cas12a-RPA	60	50 copies/μL	Tissue	[146]
*Heterodera schachtii*	Cas12a-RPA	60	10^−4^ single cysts	Tissue	[148]
*Clonorchis sinensis*	Cas12a-RPA	<60	1 copy/μL	Feces/Tissue	[147]

^a^, The time in parentheses is the time required for sample preparation or DNA extraction.

## Data Availability

Not applicable.

## Abbreviations

CRISPR: Clustered Regularly Interspaced Short Palindromic Repeats; crRNA, CRISPR RNA; DALY, disability-adjusted life year; NAAT, nucleic acid amplification test; NTD, neglected tropical disease; PAM, protospacer adjacent motif; POCT, point-of-care testing.

## References

[1] Montoya J.G., Liesenfeld O. (2004). Toxoplasmosis. Lancet.

[2] Theel E.S., Pritt B.S. (2016). Parasites. Microbiol. Spectr..

[3] Nordling L. (2023). Malaria’s modelling problem. Nature.

[4] WHO Working to Overcome the Global Impact of Neglected Tropical Diseases: First WHO Report on Neglected Tropical Diseases. https://www.who.int/publications-detail-redirect/9789241564090.

[5] The L. (2022). Neglected tropical diseases: Ending the neglect of populations. Lancet.

[6] Torgerson P.R., Devleesschauwer B., Praet N., Speybroeck N., Willingham A.L., Kasuga F., Rokni M.B., Zhou X.N., Fevre E.M., Sripa B. (2015). World Health Organization Estimates of the Global and Regional Disease Burden of 11 Foodborne Parasitic Diseases, 2010: A Data Synthesis. PLoS Med..

[7] WHO World Malaria Report 2021. https://www.who.int/teams/global-malaria-programme/reports/world-malaria-report-2021.

[8] Meinel T.R., Gottstein B., Geib V., Keel M.J., Biral R., Mohaupt M., Brügger J. (2018). Vertebral alveolar echinococcosis—A case report, systematic analysis, and review of the literature. Lancet Infect. Dis..

[9] Wong S.S., Fung K.S., Chau S., Poon R.W., Wong S.C., Yuen K.Y. (2014). Molecular diagnosis in clinical parasitology: When and why?. Exp. Biol. Med..

[10] Momcilovic S., Cantacessi C., Arsic-Arsenijevic V., Otranto D., Tasic-Otasevic S. (2019). Rapid diagnosis of parasitic diseases: Current scenario and future needs. Clin. Microbiol. Infect..

[11] Zuo X., Fan C., Chen H.-Y. (2017). Biosensing: CRISPR-powered diagnostics. Nat. Biomed. Eng..

[12] Yue H., Huang M., Tian T., Xiong E., Zhou X. (2021). Advances in Clustered, Regularly Interspaced Short Palindromic Repeats (CRISPR)-Based Diagnostic Assays Assisted by Micro/Nanotechnologies. ACS Nano.

[13] Dai Y., Wu Y., Liu G., Gooding J.J. (2020). CRISPR Mediated Biosensing Toward Understanding Cellular Biology and Point-of-Care Diagnosis. Angew. Chem. Int. Ed. Engl..

[14] Chertow D.S. (2018). Next-generation diagnostics with CRISPR. Science.

[15] Li Y., Li S., Wang J., Liu G. (2019). CRISPR/Cas Systems towards Next-Generation Biosensing. Trends Biotechnol..

[16] Huang T., Zhang R., Li J. (2023). CRISPR-Cas-based techniques for pathogen detection: Retrospect, recent advances, and future perspectives. J. Adv. Res..

[17] Singh D., Mallon J., Poddar A., Wang Y., Tippana R., Yang O., Bailey S., Ha T. (2018). Real-time observation of DNA target interrogation and product release by the RNA-guided endonuclease CRISPR Cpf1 (Cas12a). Proc. Natl. Acad. Sci. USA.

[18] Wu C., Chen Z., Li C., Hao Y., Tang Y., Yuan Y., Chai L., Fan T., Yu J., Ma X. (2022). CRISPR-Cas12a-Empowered Electrochemical Biosensor for Rapid and Ultrasensitive Detection of SARS-CoV-2 Delta Variant. Nano-Micro Lett..

[19] Wang S., Li H., Dong K., Shu W., Zhang J., Zhang J., Zhao R., Wei S., Feng D., Xiao X. (2023). A universal and specific RNA biosensor via DNA circuit-mediated PAM-independent CRISPR/Cas12a and PolyA-rolling circle amplification. Biosens. Bioelectron..

[20] van Dongen J.E., Berendsen J.T.W., Steenbergen R.D.M., Wolthuis R.M.F., Eijkel J.C.T., Segerink L.I. (2020). Point-of-care CRISPR/Cas nucleic acid detection: Recent advances, challenges and opportunities. Biosens. Bioelectron..

[21] Chen S.J., Rai C.I., Wang S.C., Chen Y.C. (2023). Point-of-Care Testing for Infectious Diseases Based on Class 2 CRISPR/Cas Technology. Diagnostics.

[22] Lu S., Tong X., Han Y., Zhang K., Zhang Y., Chen Q., Duan J., Lei X., Huang M., Qiu Y. (2022). Fast and sensitive detection of SARS-CoV-2 RNA using suboptimal protospacer adjacent motifs for Cas12a. Nat. Biomed. Eng..

[23] Lee R.A., Puig H., Nguyen P.Q., Angenent-Mari N.M., Donghia N.M., McGee J.P., Dvorin J.D., Klapperich C.M., Pollock N.R., Collins J.J. (2020). Ultrasensitive CRISPR-based diagnostic for field-applicable detection of *Plasmodium* species in symptomatic and asymptomatic malaria. Proc. Natl. Acad. Sci. USA.

[24] Lei R., Li L., Wu P., Fei X., Zhang Y., Wang J., Zhang D., Zhang Q., Yang N., Wang X. (2022). RPA/CRISPR/Cas12a-Based On-Site and Rapid Nucleic Acid Detection of *Toxoplasma gondii* in the Environment. ACS Synth. Biol..

[25] Wang X., Cheng M., Yang S., Xing C., Li Q., Zhu Y., Ji Y., Du Y. (2023). CRISPR/Cas12a combined with RPA for detection of *T. gondii* in mouse whole blood. Parasites Vectors.

[26] Li Y., Deng F., Hall T., Vesey G., Goldys E.M. (2021). CRISPR/Cas12a-powered immunosensor suitable for ultra-sensitive whole *Cryptosporidium* oocyst detection from water samples using a plate reader. Water Res..

[27] Cherkaoui D., Mesquita S.G., Huang D., Lugli E.B., Webster B.L., McKendry R.A. (2023). CRISPR-assisted test for *Schistosoma haematobium*. Sci. Rep..

[28] Daly R., Chiodini P.L. (2012). Laboratory investigations and diagnosis of tropical diseases in travelers. Infect. Dis. Clin. N. Am..

[29] Kettelhut M.M., Chiodini P.L., Edwards H., Moody A. (2003). External quality assessment schemes raise standards: Evidence from the UKNEQAS parasitology subschemes. J. Clin. Pathol..

[30] Rosenblatt J.E. (2009). Laboratory diagnosis of infections due to blood and tissue parasites. Clin. Infect. Dis..

[31] Kalogeropoulos D., Sakkas H., Mohammed B., Vartholomatos G., Malamos K., Sreekantam S., Kanavaros P., Kalogeropoulos C. (2022). Ocular toxoplasmosis: A review of the current diagnostic and therapeutic approaches. Int. Ophthalmol..

[32] Rajput R., Denniston A.K., Murray P.I. (2018). False Negative *Toxoplasma* Serology in an Immunocompromised Patient with PCR Positive Ocular Toxoplasmosis. Ocul. Immunol. Inflamm..

[33] Taylor S.C., Nadeau K., Abbasi M., Lachance C., Nguyen M., Fenrich J. (2019). The Ultimate qPCR Experiment: Producing Publication Quality, Reproducible Data the First Time. Trends Biotechnol..

[34] Park J., Lee K.G., Han D.H., Lee J.S., Lee S.J., Park J.K. (2021). Pushbutton-activated microfluidic dropenser for droplet digital PCR. Biosens. Bioelectron..

[35] Harshitha R., Arunraj D.R. (2021). Real-time quantitative PCR: A tool for absolute and relative quantification. Biochem. Mol. Biol. Educ. A Bimon. Publ. Int. Union. Biochem. Mol. Biol..

[36] Gadkar V., Filion M. (2014). New Developments in Quantitative Real-time Polymerase Chain Reaction Technology. Curr. Issues Mol. Biol..

[37] Kobets T., Badalova J., Grekov I., Havelkova H., Svobodova M., Lipoldova M. (2010). Leishmania parasite detection and quantification using PCR-ELISA. Nat. Protoc..

[38] Qian W., Yan W., Lv C., Bai R., Wang T., Wei Z., Zhang M. (2020). Molecular Detection and Genotyping of *Toxoplasma gondii* and *Neospora caninum* in Slaughtered Goats in Central China. Foodborne Pathog. Dis..

[39] Waitumbi J.N., Gerlach J., Afonina I., Anyona S.B., Koros J.N., Siangla J., Ankoudinova I., Singhal M., Watts K., Polhemus M.E. (2011). Malaria prevalence defined by microscopy, antigen detection, DNA amplification and total nucleic acid amplification in a malaria-endemic region during the peak malaria transmission season. Trop. Med. Int. Health.

[40] Alhassan A., Li Z., Poole C.B., Carlow C.K. (2015). Expanding the MDx toolbox for filarial diagnosis and surveillance. Trends Parasitol..

[41] Pomari E., Piubelli C., Perandin F., Bisoffi Z. (2019). Digital PCR: A new technology for diagnosis of parasitic infections. Clin. Microbiol. Infect..

[42] Zhao Y., Chen F., Li Q., Wang L., Fan C. (2015). Isothermal Amplification of Nucleic Acids. Chem. Rev..

[43] Lalremruata A., Nguyen T.T., McCall M.B.B., Mombo-Ngoma G., Agnandji S.T., Adegnika A.A., Lell B., Ramharter M., Hoffman S.L., Kremsner P.G. (2020). Recombinase Polymerase Amplification and Lateral Flow Assay for Ultrasensitive Detection of Low-Density *Plasmodium falciparum* Infection from Controlled Human Malaria Infection Studies and Naturally Acquired Infections. J. Clin. Microbiol..

[44] Khan M.A.A., Faisal K., Chowdhury R., Nath R., Ghosh P., Ghosh D., Hossain F., Abd El Wahed A., Mondal D. (2021). Evaluation of molecular assays to detect *Leishmania donovani* in Phlebotomus argentipes fed on post-kala-azar dermal leishmaniasis patients. Parasit. Vectors.

[45] Xu J., Rong R., Zhang H.Q., Shi C.J., Zhu X.Q., Xia C.M. (2010). Sensitive and rapid detection of *Schistosoma japonicum* DNA by loop-mediated isothermal amplification (LAMP). Int. J. Parasitol..

[46] Wang Z.H., Zhang W., Zhang X.Z., Yao X.R., Huang W., Jia H., Liu X.L., Hou S.H., Wang X.J. (2021). Development of a real-time recombinase-aided amplification (RT-RAA) molecular diagnosis assay for sensitive and rapid detection of *Toxoplasma gondii*. Vet. Parasitol..

[47] Guo Z., Xing G., Li P., Jin Q., Lu Q., Zhang G. (2023). Development and application of a recombinase-aided amplification and lateral flow assay for rapid detection of pseudorabies virus from clinical crude samples. Int. J. Biol. Macromol..

[48] Lobato I.M., O’Sullivan C.K. (2018). Recombinase polymerase amplification: Basics, applications and recent advances. Trends Anal. Chem..

[49] Shang Y., Sun J., Ye Y., Zhang J., Zhang Y., Sun X. (2020). Loop-mediated isothermal amplification-based microfluidic chip for pathogen detection. Crit. Rev. Food Sci. Nutr..

[50] Qiu M., Zhou X.M., Liu L. (2022). Improved Strategies for CRISPR-Cas12-based Nucleic Acids Detection. J. Anal. Test..

[51] Shapiro J.A., von Sternberg R. (2005). Why repetitive DNA is essential to genome function. Biol. Rev. Camb. Philos. Soc..

[52] Zheng H., Zhang W., Zhang L., Zhang Z., Li J., Lu G., Zhu Y., Wang Y., Huang Y., Liu J. (2013). The genome of the hydatid tapeworm *Echinococcus granulosus*. Nat. Genet..

[53] Gonzalez A., Prediger E., Huecas M.E., Nogueira N., Lizardi P.M. (1984). Minichromosomal repetitive DNA in *Trypanosoma cruzi*: Its use in a high-sensitivity parasite detection assay. Proc. Natl. Acad. Sci. USA.

[54] Homan W.L., Vercammen M., De Braekeleer J., Verschueren H. (2000). Identification of a 200- to 300-fold repetitive 529 bp DNA fragment in *Toxoplasma gondii*, and its use for diagnostic and quantitative PCR. Int. J. Parasitol..

[55] Demas A., Oberstaller J., DeBarry J., Lucchi N.W., Srinivasamoorthy G., Sumari D., Kabanywanyi A.M., Villegas L., Escalante A.A., Kachur S.P. (2011). Applied genomics: Data mining reveals species-specific malaria diagnostic targets more sensitive than 18S rRNA. J. Clin. Microbiol..

[56] Singh R., Singh D.P., Gupta R., Savargaonkar D., Singh O.P., Nanda N., Bhatt R.M., Valecha N. (2014). Comparison of three PCR-based assays for the non-invasive diagnosis of malaria: Detection of *Plasmodium* parasites in blood and saliva. Eur. J. Clin. Microbiol. Infect. Dis. Off. Publ. Eur. Soc. Clin. Microbiol..

[57] Azam M., Upmanyu K., Gupta R., Sruthy K.S., Matlani M., Savargaonkar D., Singh R. (2021). Development of Two-Tube Loop-Mediated Isothermal Amplification Assay for Differential Diagnosis of *Plasmodium falciparum* and *Plasmodium vivax* and Its Comparison with Loopamp™ Malaria. Diagnostics.

[58] Rosenzvit M.C., Canova S.G., Kamenetzky L., Ledesma B.A., Guarnera E.A. (1997). *Echinococcus granulosus*: Cloning and characterization of a tandemly repeated DNA element. Exp. Parasitol..

[59] Abbasi I., Hamburger J., Raoul F., Craig P.S., Campos-Ponce M., Branzburg A., Hafez S.K.A. (2003). Copro-Diagnosis of *Echinococcus granulosus* Infection in Dogs by Amplification of a Newly Identified Repeated DNA Sequence. Am. J. Trop. Med. Hyg..

[60] Chapman A., Vallejo V., Mossie K.G., Ortiz D., Agabian N., Flisser A. (1995). Isolation and characterization of species-specific DNA probes from *Taenia solium* and *Taenia saginata* and their use in an egg detection assay. J. Clin. Microbiol..

[61] González L.M., Montero E., Harrison L.J., Parkhouse R.M., Garate T. (2000). Differential diagnosis of *Taenia saginata* and *Taenia solium* infection by PCR. J. Clin. Microbiol..

[62] Hamburger J., Turetski T., Kapeller I., Deresiewicz R. (1991). Highly repeated short DNA sequences in the genome of *Schistosoma mansoni* recognized by a species-specific probe. Mol. Biochem. Parasitol..

[63] Mwangi I.N., Agola E.L., Mugambi R.M., Shiraho E.A., Mkoji G.M. (2018). Development and Evaluation of a Loop-Mediated Isothermal Amplification Assay for Diagnosis of *Schistosoma mansoni* Infection in Faecal Samples. J. Parasitol. Res..

[64] Hamburger J., Xu Y.X., Ramzy R.M., Jourdane J., Ruppel A. (1998). Development and laboratory evaluation of a polymerase chain reaction for monitoring *Schistosoma mansoni* infestation of water. Am. J. Trop. Med. Hyg..

[65] Hamburger J., He N., Abbasi I., Ramzy R.M., Jourdane J., Ruppel A. (2001). Polymerase chain reaction assay based on a highly repeated sequence of *Schistosoma haematobium*: A potential tool for monitoring schistosome-infested water. Am. J. Trop. Med. Hyg..

[66] Hertel J., Hamburger J., Haberl B., Haas W. (2002). Detection of bird schistosomes in lakes by PCR and filter-hybridization. Exp. Parasitol..

[67] Lodh N., Caro R., Sofer S., Scott A., Krolewiecki A., Shiff C. (2016). Diagnosis of *Strongyloides stercoralis*: Detection of parasite-derived DNA in urine. Acta Trop..

[68] McReynolds L.A., DeSimone S.M., Williams S.A. (1986). Cloning and comparison of repeated DNA sequences from the human filarial parasite *Brugia malayi* and the animal parasite *Brugia pahangi*. Proc. Natl. Acad. Sci. USA.

[69] Albers A., Sartono E., Wahyuni S., Yazdanbakhsh M., Maizels R.M., Klarmann-Schulz U., Pfarr K., Hoerauf A. (2014). Real-time PCR detection of the HhaI tandem DNA repeat in pre- and post-patent *Brugia malayi* Infections: A study in Indonesian transmigrants. Parasit. Vectors.

[70] Zhong M., McCarthy J., Bierwert L., Lizotte-Waniewski M., Chanteau S., Nutman T.B., Ottesen E.A., Williams S.A. (1996). A polymerase chain reaction assay for detection of the parasite *Wuchereria bancrofti* in human blood samples. Am. J. Trop. Med. Hyg..

[71] Rao R.U., Atkinson L.J., Ramzy R.M., Helmy H., Farid H.A., Bockarie M.J., Susapu M., Laney S.J., Williams S.A., Weil G.J. (2006). A real-time PCR-based assay for detection of *Wuchereria bancrofti* DNA in blood and mosquitoes. Am. J. Trop. Med. Hyg..

[72] Saul A., Yeganeh F., Howard R.J. (1992). Cloning and characterization of a novel multicopy, repetitive sequence of *Plasmodium falciparum*, REP51. Immunol. Cell Biol..

[73] Hotterbeekx A., Raimon S., Abd-Elfarag G., Carter J.Y., Sebit W., Suliman A., Siewe Fodjo J.N., De Witte P., Logora M.Y., Colebunders R. (2020). *Onchocerca volvulus* is not detected in the cerebrospinal fluid of persons with onchocerciasis-associated epilepsy. Int. J. Infect. Dis..

[74] Macfarlane C.L., Quek S., Pionnier N., Turner J.D., Wanji S., Wagstaff S.C., Taylor M.J. (2020). The insufficiency of circulating miRNA and DNA as diagnostic tools or as biomarkers of treatment efficacy for *Onchocerca volvulus*. Sci. Rep..

[75] Williams K.M., Fessler M.K., Bloomfield R.A., Sandke W.D., Malekshahi C.R., Keroack C.D., Duignan P.J., Torquato S.D., Williams S.A. (2020). A novel quantitative real-time PCR diagnostic assay for fecal and nasal swab detection of an otariid lungworm, *Parafilaroides decorus*. Int. J. Parasitol. Parasites Wildl..

[76] Luppa P.B., Müller C., Schlichtiger A., Schlebusch H. (2011). Point-of-care testing (POCT): Current techniques and future perspectives. Trends Anal. Chem..

[77] Ishino Y., Shinagawa H., Makino K., Amemura M., Nakata A. (1987). Nucleotide sequence of the iap gene, responsible for alkaline phosphatase isozyme conversion in *Escherichia coli*, and identification of the gene product. J. Bacteriol..

[78] Jansen R., Embden J.D., Gaastra W., Schouls L.M. (2002). Identification of genes that are associated with DNA repeats in prokaryotes. Mol. Microbiol..

[79] Mohanraju P., Makarova K.S., Zetsche B., Zhang F., Koonin E.V., van der Oost J. (2016). Diverse evolutionary roots and mechanistic variations of the CRISPR-Cas systems. Science.

[80] Jinek M., Chylinski K., Fonfara I., Hauer M., Doudna J.A., Charpentier E. (2012). A programmable dual-RNA-guided DNA endonuclease in adaptive bacterial immunity. Science.

[81] East-Seletsky A., O’Connell M.R., Burstein D., Knott G.J., Doudna J.A. (2017). RNA Targeting by Functionally Orthogonal Type VI-A CRISPR-Cas Enzymes. Mol. Cell.

[82] Chen J.S., Ma E.B., Harrington L.B., Da Costa M., Tian X.R., Palefsky J.M., Doudna J.A. (2018). CRISPR-Cas12a target binding unleashes indiscriminate single-stranded DNase activity. Science.

[83] East-Seletsky A., O’Connell M.R., Knight S.C., Burstein D., Cate J.H., Tjian R., Doudna J.A. (2016). Two distinct RNase activities of CRISPR-C2c2 enable guide-RNA processing and RNA detection. Nature.

[84] Li S.Y., Cheng Q.X., Wang J.M., Li X.Y., Zhang Z.L., Gao S., Cao R.B., Zhao G.P., Wang J. (2018). CRISPR-Cas12a-assisted nucleic acid detection. Cell Discov..

[85] Hsu P.D., Lander E.S., Zhang F. (2014). Development and applications of CRISPR-Cas9 for genome engineering. Cell.

[86] Ran F.A., Cong L., Yan W.X., Scott D.A., Gootenberg J.S., Kriz A.J., Zetsche B., Shalem O., Wu X., Makarova K.S. (2015). In vivo genome editing using *Staphylococcus aureus* Cas9. Nature.

[87] Pardee K., Green A.A., Takahashi M.K., Braff D., Lambert G., Lee J.W., Ferrante T., Ma D., Donghia N., Fan M. (2016). Rapid, Low-Cost Detection of Zika Virus Using Programmable Biomolecular Components. Cell.

[88] Zhang H., Qin C., An C., Zheng X., Wen S., Chen W., Liu X., Lv Z., Yang P., Xu W. (2021). Application of the CRISPR/Cas9-based gene editing technique in basic research, diagnosis, and therapy of cancer. Mol. Cancer.

[89] Gootenberg J.S., Abudayyeh O.O., Lee J.W., Essletzbichler P., Dy A.J., Joung J., Verdine V., Donghia N., Daringer N.M., Freije C.A. (2017). Nucleic acid detection with CRISPR-Cas13a/C2c2. Science.

[90] Shmakov S., Smargon A., Scott D., Cox D., Pyzocha N., Yan W., Abudayyeh O.O., Gootenberg J.S., Makarova K.S., Wolf Y.I. (2017). Diversity and evolution of class 2 CRISPR-Cas systems. Nat. Rev. Microbiol..

[91] Zetsche B., Gootenberg J.S., Abudayyeh O.O., Slaymaker I.M., Makarova K.S., Essletzbichler P., Volz S.E., Joung J., van der Oost J., Regev A. (2015). Cpf1 is a single RNA-guided endonuclease of a class 2 CRISPR-Cas system. Cell.

[92] Shmakov S., Abudayyeh O.O., Makarova K.S., Wolf Y.I., Gootenberg J.S., Semenova E., Minakhin L., Joung J., Konermann S., Severinov K. (2015). Discovery and Functional Characterization of Diverse Class 2 CRISPR-Cas Systems. Mol. Cell.

[93] Swarts D.C., Jinek M. (2019). Mechanistic Insights into the cis- and trans-Acting DNase Activities of Cas12a. Mol. Cell.

[94] Yamano T., Nishimasu H., Zetsche B., Hirano H., Slaymaker I.M., Li Y., Fedorova I., Nakane T., Makarova K.S., Koonin E.V. (2016). Crystal Structure of Cpf1 in Complex with Guide RNA and Target DNA. Cell.

[95] Mann J.G., Pitts R.J. (2022). PrimedSherlock: A tool for rapid design of highly specific CRISPR-Cas12 crRNAs. BMC Bioinform..

[96] Zetsche B., Heidenreich M., Mohanraju P., Fedorova I., Kneppers J., DeGennaro E.M., Winblad N., Choudhury S.R., Abudayyeh O.O., Gootenberg J.S. (2017). Multiplex gene editing by CRISPR-Cpf1 using a single crRNA array. Nat. Biotechnol..

[97] Tang X., Lowder L.G., Zhang T., Malzahn A.A., Zheng X., Voytas D.F., Zhong Z., Chen Y., Ren Q., Li Q. (2017). A CRISPR-Cpf1 system for efficient genome editing and transcriptional repression in plants. Nat. Plants.

[98] Mustafa M.I., Makhawi A.M. (2021). SHERLOCK and DETECTR: CRISPR-Cas Systems as Potential Rapid Diagnostic Tools for Emerging Infectious Diseases. J. Clin. Microbiol..

[99] Kostyusheva A., Brezgin S., Babin Y., Vasilyeva I., Glebe D., Kostyushev D., Chulanov V. (2022). CRISPR-Cas systems for diagnosing infectious diseases. Methods.

[100] Li H., Cui X., Sun L., Deng X., Liu S., Zou X., Li B., Wang C., Wang Y., Liu Y. (2021). High concentration of Cas12a effector tolerates more mismatches on ssDNA. FASEB J. Off. Publ. Fed. Am. Soc. Exp. Biol..

[101] Li S.Y., Cheng Q.X., Liu J.K., Nie X.Q., Zhao G.P., Wang J. (2018). CRISPR-Cas12a has both cis- and trans-cleavage activities on single-stranded DNA. Cell Res..

[102] Strohkendl I., Saifuddin F.A., Rybarski J.R., Finkelstein I.J., Russell R. (2018). Kinetic Basis for DNA Target Specificity of CRISPR-Cas12a. Mol. Cell.

[103] Nalefski E.A., Patel N., Leung P.J.Y., Islam Z., Kooistra R.M., Parikh I., Marion E., Knott G.J., Doudna J.A., Le Ny A.M. (2021). Kinetic analysis of Cas12a and Cas13a RNA-Guided nucleases for development of improved CRISPR-Based diagnostics. iScience.

[104] Dai Y., Somoza R.A., Wang L., Welter J.F., Li Y., Caplan A.I., Liu C.C. (2019). Exploring the Trans-Cleavage Activity of CRISPR-Cas12a (cpf1) for the Development of a Universal Electrochemical Biosensor. Angew. Chem. Int. Ed. Engl..

[105] Li Y., Mansour H., Watson C.J.F., Tang Y., MacNeil A.J., Li F. (2021). Amplified detection of nucleic acids and proteins using an isothermal proximity CRISPR Cas12a assay. Chem. Sci..

[106] Liang M., Li Z., Wang W., Liu J., Liu L., Zhu G., Karthik L., Wang M., Wang K.F., Wang Z. (2019). A CRISPR-Cas12a-derived biosensing platform for the highly sensitive detection of diverse small molecules. Nat. Commun..

[107] Shi K., Xie S., Tian R., Wang S., Lu Q., Gao D., Lei C., Zhu H., Nie Z. (2021). A CRISPR-Cas autocatalysis-driven feedback amplification network for supersensitive DNA diagnostics. Sci. Adv..

[108] Xiong Y., Zhang J., Yang Z., Mou Q., Ma Y., Xiong Y., Lu Y. (2020). Functional DNA Regulated CRISPR-Cas12a Sensors for Point-of-Care Diagnostics of Non-Nucleic-Acid Targets. J. Am. Chem. Soc..

[109] Wang S.Y., Du Y.C., Wang D.X., Ma J.Y., Tang A.N., Kong D.M. (2021). Signal amplification and output of CRISPR/Cas-based biosensing systems: A review. Anal. Chim. Acta.

[110] Liu H., Wang J., Zeng H., Liu X., Jiang W., Wang Y., Ouyang W., Tang X. (2021). RPA-Cas12a-FS: A frontline nucleic acid rapid detection system for food safety based on CRISPR-Cas12a combined with recombinase polymerase amplification. Food Chem..

[111] Broughton J.P., Deng X., Yu G., Fasching C.L., Servellita V., Singh J., Miao X., Streithorst J.A., Granados A., Sotomayor-Gonzalez A. (2020). CRISPR-Cas12-based detection of SARS-CoV-2. Nat. Biotechnol..

[112] Yu F., Zhang K., Wang Y., Li D., Cui Z., Huang J., Zhang S., Li X., Zhang L. (2021). CRISPR/Cas12a-based on-site diagnostics of *Cryptosporidium parvum* IId-subtype-family from human and cattle fecal samples. Parasit. Vectors.

[113] Hu M., Zhu D., Zhou X. (2022). M-CDC: Magnetic pull-down-assisted colorimetric method based on the CRISPR/Cas12a system. Methods.

[114] Wang R., Qian C., Pang Y., Li M., Yang Y., Ma H., Zhao M., Qian F., Yu H., Liu Z. (2021). opvCRISPR: One-pot visual RT-LAMP-CRISPR platform for SARS-cov-2 detection. Biosens. Bioelectron..

[115] Wang X., Zhong M., Liu Y., Ma P., Dang L., Meng Q., Wan W., Ma X., Liu J., Yang G. (2020). Rapid and sensitive detection of COVID-19 using CRISPR/Cas12a-based detection with naked eye readout, CRISPR/Cas12a-NER. Sci. Bull..

[116] Chen F.E., Lee P.W., Trick A.Y., Park J.S., Chen L., Shah K., Mostafa H., Carroll K.C., Hsieh K., Wang T.H. (2021). Point-of-care CRISPR-Cas-assisted SARS-CoV-2 detection in an automated and portable droplet magnetofluidic device. Biosens. Bioelectron..

[117] Ding X., Yin K., Li Z., Lalla R.V., Ballesteros E., Sfeir M.M., Liu C. (2020). Ultrasensitive and visual detection of SARS-CoV-2 using all-in-one dual CRISPR-Cas12a assay. Nat. Commun..

[118] Xiao H., Hu J., Huang C., Feng W., Liu Y., Kumblathan T., Tao J., Xu J., Le X.C., Zhang H. (2023). CRISPR techniques and potential for the detection and discrimination of SARS-CoV-2 variants of concern. Trends Anal. Chem..

[119] Tian T., Shu B., Jiang Y., Ye M., Liu L., Guo Z., Han Z., Wang Z., Zhou X. (2021). An Ultralocalized Cas13a Assay Enables Universal and Nucleic Acid Amplification-Free Single-Molecule RNA Diagnostics. ACS Nano.

[120] Ding X., Yin K., Li Z., Sfeir M.M., Liu C. (2021). Sensitive quantitative detection of SARS-CoV-2 in clinical samples using digital warm-start CRISPR assay. Biosens. Bioelectron..

[121] Moon J., Liu C. (2023). Asymmetric CRISPR enabling cascade signal amplification for nucleic acid detection by competitive crRNA. Nat. Commun..

[122] Lee I., Kwon S.J., Sorci M., Heeger P.S., Dordick J.S. (2021). Highly Sensitive Immuno-CRISPR Assay for CXCL9 Detection. Anal. Chem..

[123] Tang Y., Song T., Gao L., Yin S., Ma M., Tan Y., Wu L., Yang Y., Wang Y., Lin T. (2022). A CRISPR-based ultrasensitive assay detects attomolar concentrations of SARS-CoV-2 antibodies in clinical samples. Nat. Commun..

[124] Cheng M., Xiong E., Tian T., Zhu D., Ju H.Q., Zhou X. (2021). A CRISPR-driven colorimetric code platform for highly accurate telomerase activity assay. Biosens. Bioelectron..

[125] Tian T., Zhou X. (2023). CRISPR-Based Biosensing Strategies: Technical Development and Application Prospects. Annu. Rev. Anal. Chem..

[126] Sam I.K., Chen Y.Y., Ma J., Li S.Y., Ying R.Y., Li L.X., Ji P., Wang S.J., Xu J., Bao Y.J. (2021). TB-QUICK: CRISPR-Cas12b-assisted rapid and sensitive detection of *Mycobacterium tuberculosis*. J. Infect..

[127] Kachwala M.J., Smith C.W., Nandu N., Yigit M.V. (2021). Reprogrammable Gel Electrophoresis Detection Assay Using CRISPR-Cas12a and Hybridization Chain Reaction. Anal. Chem..

[128] Tian T., Qiu Z., Jiang Y., Zhu D., Zhou X. (2022). Exploiting the orthogonal CRISPR-Cas12a/Cas13a trans-cleavage for dual-gene virus detection using a handheld device. Biosens. Bioelectron..

[129] Dincer C., Bruch R., Kling A., Dittrich P.S., Urban G.A. (2017). Multiplexed Point-of-Care Testing-xPOCT. Trends Biotechnol..

[130] Gootenberg J.S., Abudayyeh O.O., Kellner M.J., Joung J., Collins J.J., Zhang F. (2018). Multiplexed and portable nucleic acid detection platform with Cas13, Cas12a, and Csm6. Science.

[131] Li Y., Liu L., Liu G. (2019). CRISPR/Cas Multiplexed Biosensing: A Challenge or an Insurmountable Obstacle?. Trends Biotechnol..

[132] Shao N., Han X., Song Y., Zhang P., Qin L. (2019). CRISPR-Cas12a Coupled with Platinum Nanoreporter for Visual Quantification of SNVs on a Volumetric Bar-Chart Chip. Anal. Chem..

[133] Zhao R., Tang Y., Song D., Liu M., Li B. (2023). CRISPR/Cas12a-Responsive Hydrogels for Conjugation-Free and Universal Indicator Release in Colorimetric Detection. Anal. Chem..

[134] Kosack C.S., Page A.L., Klatser P.R. (2017). A guide to aid the selection of diagnostic tests. Bull. World Health Organ..

[135] He Q., Yu D., Bao M., Korensky G., Chen J., Shin M., Kim J., Park M., Qin P., Du K. (2020). High-throughput and all-solution phase African Swine Fever Virus (ASFV) detection using CRISPR-Cas12a and fluorescence based point-of-care system. Biosens. Bioelectron..

[136] Wei H., Li J., Liu Y., Cheng W., Huang H., Liang X., Huang W., Lin L., Zheng Y., Chen W. (2023). Rapid and Ultrasensitive Detection of *Plasmodium* spp. Parasites via the RPA-CRISPR/Cas12a Platform. ACS Infect. Dis..

[137] Bai J., Lin H., Li H., Zhou Y., Liu J., Zhong G., Wu L., Jiang W., Du H., Yang J. (2019). Cas12a-Based On-Site and Rapid Nucleic Acid Detection of African Swine Fever. Front. Microbiol..

[138] Mukama O., Yuan T., He Z., Li Z., Habimana J.d.D., Hussain M., Li W., Yi Z., Liang Q., Zeng L. (2020). A high fidelity CRISPR/Cas12a based lateral flow biosensor for the detection of HPV16 and HPV18. Sens. Actuators B Chem..

[139] Tsou J.H., Leng Q., Jiang F. (2019). A CRISPR Test for Detection of Circulating Nuclei Acids. Transl. Oncol..

[140] Yuan C., Tian T., Sun J., Hu M., Wang X., Xiong E., Cheng M., Bao Y., Lin W., Jiang J. (2020). Universal and Naked-Eye Gene Detection Platform Based on the Clustered Regularly Interspaced Short Palindromic Repeats/Cas12a/13a System. Anal. Chem..

[141] Zhang D., Yan Y., Que H., Yang T., Cheng X., Ding S., Zhang X., Cheng W. (2020). CRISPR/Cas12a-Mediated Interfacial Cleaving of Hairpin DNA Reporter for Electrochemical Nucleic Acid Sensing. ACS Sens..

[142] English M.A., Soenksen L.R., Gayet R.V., de Puig H., Angenent-Mari N.M., Mao A.S., Nguyen P.Q., Collins J.J. (2019). Programmable CRISPR-responsive smart materials. Science.

[143] WHO Malaria Rapid Diagnostic Test Performance: Summary Results of WHO Product Testing of Malaria RDTs: Round 8 (2016–2018). https://www.who.int/publications/i/item/9789241514965.

[144] Ma Q.N., Wang M., Zheng L.B., Lin Z.Q., Ehsan M., Xiao X.X., Zhu X.Q. (2021). RAA-Cas12a-Tg: A Nucleic Acid Detection System for *Toxoplasma gondii* Based on CRISPR-Cas12a Combined with Recombinase-Aided Amplification (RAA). Microorganisms.

[145] Galvani A.T., Christ A.P.G., Padula J.A., Barbosa M.R.F., de Araújo R.S., Sato M.I.Z., Razzolini M.T.P. (2019). Real-time PCR detection of *Toxoplasma gondii* in surface water samples in São Paulo, Brazil. Parasitol. Res..

[146] Kanitchinda S., Srisala J., Suebsing R., Prachumwat A., Chaijarasphong T. (2020). CRISPR-Cas fluorescent cleavage assay coupled with recombinase polymerase amplification for sensitive and specific detection of *Enterocytozoon hepatopenaei*. Biotechnol. Rep..

[147] Huang T., Li L., Li J., Li X., Li S., Wang X., Zhang N., Yu Y., Zhang X., Zhao Z. (2023). Rapid, sensitive, and visual detection of *Clonorchis sinensis* with an RPA-CRISPR/Cas12a-based dual readout portable platform. Int. J. Biol. Macromol..

[148] Yao K., Peng D., Jiang C., Zhao W., Li G., Huang W., Kong L., Gao H., Zheng J., Peng H. (2021). Rapid and Visual Detection of *Heterodera schachtii* Using Recombinase Polymerase Amplification Combined with Cas12a-Mediated Technology. Int. J. Mol. Sci..

[149] Fleming K.A., Horton S., Wilson M.L., Atun R., DeStigter K., Flanigan J., Sayed S., Adam P., Aguilar B., Andronikou S. (2021). The Lancet Commission on diagnostics: Transforming access to diagnostics. Lancet.

[150] Huyke D.A., Ramachandran A., Bashkirov V.I., Kotseroglou E.K., Kotseroglou T., Santiago J.G. (2022). Enzyme Kinetics and Detector Sensitivity Determine Limits of Detection of Amplification-Free CRISPR-Cas12 and CRISPR-Cas13 Diagnostics. Anal. Chem..

[151] Ramachandran A., Santiago J.G. (2021). CRISPR Enzyme Kinetics for Molecular Diagnostics. Anal. Chem..

[152] Garneau J.E., Dupuis M., Villion M., Romero D.A., Barrangou R., Boyaval P., Fremaux C., Horvath P., Magadán A.H., Moineau S. (2010). The CRISPR/Cas bacterial immune system cleaves bacteriophage and plasmid DNA. Nature.

[153] Dong D., Ren K., Qiu X., Zheng J., Guo M., Guan X., Liu H., Li N., Zhang B., Yang D. (2016). The crystal structure of Cpf1 in complex with CRISPR RNA. Nature.

[154] Gao P., Yang H., Rajashankar K.R., Huang Z., Patel D.J. (2016). Type V CRISPR-Cas Cpf1 endonuclease employs a unique mechanism for crRNA-mediated target DNA recognition. Cell Res..

[155] Yamano T., Zetsche B., Ishitani R., Zhang F., Nishimasu H., Nureki O. (2017). Structural Basis for the Canonical and Non-canonical PAM Recognition by CRISPR-Cpf1. Mol. Cell.

[156] Kim H.K., Song M., Lee J., Menon A.V., Jung S., Kang Y.M., Choi J.W., Woo E., Koh H.C., Nam J.W. (2017). In vivo high-throughput profiling of CRISPR-Cpf1 activity. Nat. Methods.

[157] Tong X., Zhang K., Han Y., Li T., Duan M., Ji R., Wang X., Zhou X., Zhang Y., Yin H. (2024). Fast and sensitive CRISPR detection by minimized interference of target amplification. Nat. Chem. Biol..

[158] Corsi G.I., Qu K., Alkan F., Pan X., Luo Y., Gorodkin J. (2022). CRISPR/Cas9 gRNA activity depends on free energy changes and on the target PAM context. Nat. Commun..

[159] Chen Q., Chuai G., Zhang H., Tang J., Duan L., Guan H., Li W., Li W., Wen J., Zuo E. (2023). Genome-wide CRISPR off-target prediction and optimization using RNA-DNA interaction fingerprints. Nat. Commun..

[160] Li Q., Song Z.L., Zhang Y., Zhu L., Yang Q., Liu X., Sun X., Chen X., Kong R., Fan G.C. (2023). Synergistic Incorporation of Two ssDNA Activators Enhances the Trans-Cleavage of CRISPR/Cas12a. Anal. Chem..

[161] Jiang Y., Qian X., Zheng M., Deng K., Li C. (2023). Enhancement and inactivation effect of CRISPR/Cas12a via extending hairpin activators for detection of transcription factors. Mikrochim. Acta.

[162] Kocak D.D., Josephs E.A., Bhandarkar V., Adkar S.S., Kwon J.B., Gersbach C.A. (2019). Increasing the specificity of CRISPR systems with engineered RNA secondary structures. Nat. Biotechnol..

[163] Chen Y., Mei Y., Zhao X., Jiang X. (2020). Reagents-Loaded, Automated Assay that Integrates Recombinase-Aided Amplification and Cas12a Nucleic Acid Detection for a Point-of-Care Test. Anal. Chem..

[164] Zhuo C., Zhang J., Lee J.-H., Jiao J., Cheng D., Liu L., Kim H.-W., Tao Y., Li M. (2021). Spatiotemporal control of CRISPR/Cas9 gene editing. Signal Transduct. Target. Ther..

[165] Zhou W., Brown W., Bardhan A., Delaney M., Ilk A.S., Rauen R.R., Kahn S.I., Tsang M., Deiters A. (2020). Spatiotemporal Control of CRISPR/Cas9 Function in Cells and Zebrafish using Light-Activated Guide RNA. Angew. Chem. Int. Ed..

[166] Jain P.K., Ramanan V., Schepers A.G., Dalvie N.S., Panda A., Fleming H.E., Bhatia S.N. (2016). Development of Light-Activated CRISPR Using Guide RNAs with Photocleavable Protectors. Angew. Chem. Int. Ed. Engl..

[167] Hu M., Qiu Z., Bi Z., Tian T., Jiang Y., Zhou X. (2022). Photocontrolled crRNA activation enables robust CRISPR-Cas12a diagnostics. Proc. Natl. Acad. Sci. USA.

[168] Hu M., Liu R., Qiu Z., Cao F., Tian T., Lu Y., Jiang Y., Zhou X. (2023). Light-Start CRISPR-Cas12a Reaction with Caged crRNA Enables Rapid and Sensitive Nucleic Acid Detection. Angew. Chem. Int. Ed..

[169] Pang B., Xu J., Liu Y., Peng H., Feng W., Cao Y., Wu J., Xiao H., Pabbaraju K., Tipples G. (2020). Isothermal Amplification and Ambient Visualization in a Single Tube for the Detection of SARS-CoV-2 Using Loop-Mediated Amplification and CRISPR Technology. Anal. Chem..

[170] Chen Y., Shi Y., Chen Y., Yang Z., Wu H., Zhou Z., Li J., Ping J., He L., Shen H. (2020). Contamination-free visual detection of SARS-CoV-2 with CRISPR/Cas12a: A promising method in the point-of-care detection. Biosens. Bioelectron..

[171] Jiao J., Liu Y., Yang M., Zheng J., Liu C., Ye W., Song S., Bai T., Song C., Wang M. (2023). The engineered CRISPR-Mb2Cas12a variant enables sensitive and fast nucleic acid-based pathogens diagnostics in the field. Plant Biotechnol. J..

[172] Zhang X., Guo B., Yang L., Zhao C., Wang Y., Tang Y., Yang G., Wang P., Gao S. (2023). CRISPR/Cas12a combined with recombinase polymerase amplification for rapid and sensitive detection of *Vibrio vulnificus* in one tube. Acta Biochim. Biophys. Sin..

[173] Shebanova R., Nikitchina N., Shebanov N., Mekler V., Kuznedelov K., Ulashchik E., Vasilev R., Sharko O., Shmanai V., Tarassov I. (2022). Efficient target cleavage by Type V Cas12a effectors programmed with split CRISPR RNA. Nucleic Acids Res..

[174] Nguyen L.T., Smith B.M., Jain P.K. (2020). Enhancement of trans-cleavage activity of Cas12a with engineered crRNA enables amplified nucleic acid detection. Nat. Commun..

[175] Ooi K.H., Liu M.M., Tay J.W.D., Teo S.Y., Kaewsapsak P., Jin S., Lee C.K., Hou J., Maurer-Stroh S., Lin W. (2021). An engineered CRISPR-Cas12a variant and DNA-RNA hybrid guides enable robust and rapid COVID-19 testing. Nat. Commun..

[176] Li Z., Zhao W., Ma S., Li Z., Yao Y., Fei T. (2021). A chemical-enhanced system for CRISPR-Based nucleic acid detection. Biosens. Bioelectron..

[177] Hsieh K., Zhao G., Wang T.H. (2020). Applying biosensor development concepts to improve preamplification-free CRISPR/Cas12a-Dx. Analyst.

[178] Ma P., Meng Q., Sun B., Zhao B., Dang L., Zhong M., Liu S., Xu H., Mei H., Liu J. (2020). MeCas12a, a Highly Sensitive and Specific System for COVID-19 Detection. Adv. Sci..

[179] Yue H., Shu B., Tian T., Xiong E., Huang M., Zhu D., Sun J., Liu Q., Wang S., Li Y. (2021). Droplet Cas12a Assay Enables DNA Quantification from Unamplified Samples at the Single-Molecule Level. Nano Lett..

[180] Lv H., Wang J., Zhang J., Chen Y., Yin L., Jin D., Gu D., Zhao H., Xu Y., Wang J. (2021). Definition of CRISPR Cas12a T rans-Cleavage Units to Facilitate CRISPR Diagnostics. Front. Microbiol..

[181] Rossetti M., Merlo R., Bagheri N., Moscone D., Valenti A., Saha A., Arantes P.R., Ippodrino R., Ricci F., Treglia I. (2022). Enhancement of CRISPR/Cas12a trans-cleavage activity using hairpin DNA reporters. Nucleic Acids Res..

[182] Li T., Hu R., Xia J., Xu Z., Chen D., Xi J., Liu B.F., Zhu J., Li Y., Yang Y. (2021). G-triplex: A new type of CRISPR-Cas12a reporter enabling highly sensitive nucleic acid detection. Biosens. Bioelectron..

[183] Paul R., Ostermann E., Wei Q. (2020). Advances in point-of-care nucleic acid extraction technologies for rapid diagnosis of human and plant diseases. Biosens. Bioelectron..

[184] Li H., Xie Y., Chen F., Bai H., Xiu L., Zhou X., Guo X., Hu Q., Yin K. (2023). Amplification-free CRISPR/Cas detection technology: Challenges, strategies, and perspectives. Chem. Soc. Rev..

[185] Jamshidi M.B., Lalbakhsh A., Talla J., Peroutka Z., Hadjilooei F., Lalbakhsh P., Jamshidi M., Spada L., Mirmozafari M., Dehghani M. (2020). Artificial Intelligence and COVID-19: Deep Learning Approaches for Diagnosis and Treatment. IEEE Access Pract. Innov. Open Solut..

[186] Wang B., Li Y., Zhou M., Han Y., Zhang M., Gao Z., Liu Z., Chen P., Du W., Zhang X. (2023). Smartphone-based platforms implementing microfluidic detection with image-based artificial intelligence. Nat. Commun..

[187] Bhat A.A., Nisar S., Mukherjee S., Saha N., Yarravarapu N., Lone S.N., Masoodi T., Chauhan R., Maacha S., Bagga P. (2022). Integration of CRISPR/Cas9 with artificial intelligence for improved cancer therapeutics. J. Transl. Med..

[188] Gonatopoulos-Pournatzis T., Aregger M., Brown K.R., Farhangmehr S., Braunschweig U., Ward H.N., Ha K.C.H., Weiss A., Billmann M., Durbic T. (2020). Genetic interaction mapping and exon-resolution functional genomics with a hybrid Cas9-Cas12a platform. Nat. Biotechnol..

[189] Baisya D., Ramesh A., Schwartz C., Lonardi S., Wheeldon I. (2022). Genome-wide functional screens enable the prediction of high activity CRISPR-Cas9 and -Cas12a guides in Yarrowia lipolytica. Nat. Commun..

